# Whose Responsibility Is It Anyway? Exploring Barriers to Prevention of Oral Diseases across Europe

**DOI:** 10.1177/2380084420926972

**Published:** 2020-05-21

**Authors:** H. Leggett, J. Csikar, K. Vinall-Collier, G.V.A. Douglas

**Affiliations:** 1 School of Dentistry, University of Leeds, Leeds, UK

**Keywords:** oral health, dental public health, qualitative, preventive dentistry, health services research, European Union

## Abstract

**Introduction:**

Dental caries, gum disease, and tooth loss are all preventable conditions. However, many dental care systems remain treatment oriented rather than prevention oriented. This promotes the treatment of oral diseases over preventive treatments and advice. Exploring barriers to prevention and understanding the requirements of a paradigm shift are the first steps toward delivering quality prevention-focused health care.

**Objectives:**

To qualitatively explore perceived barriers and facilitators to oral disease prevention from a multistakeholder perspective across 6 European countries.

**Methods:**

A total of 58 interviews and 13 focus groups were undertaken involving 149 participants from the United Kingdom, Denmark, Germany, the Netherlands, Ireland, and Hungary. Interviews and focus groups were conducted in each country in its native language between March 2016 and September 2017. Participants were patients (*n* = 50), dental team members (*n* = 39), dental policy makers(*n* = 33), and dental insurers (*n* = 27). The audio was transcribed, translated, and analyzed via deductive thematic analysis.

**Results:**

Five broad themes emerged that were both barriers and facilitators: dental regulation, who provides prevention, knowledge and motivation, trust, and person-level factors. Each theme was touched on in all countries; however, cross-country differences were evident surrounding the magnitude of each theme.

**Conclusion:**

Despite the different strengths and weaknesses among the systems, those who deliver, organize, and utilize each system experience similar barriers to prevention. The findings suggest that across all 6 countries, prevention in oral health care is hindered by a complex interplay of factors, with no particular dental health system offering overall greater user satisfaction. Underlying the themes were sentiments of blame, whereby each group appeared to shift responsibility for prevention to other groups. To bring about change, greater teamwork is needed in the commissioning of prevention to engender its increased value by all stakeholders within the dental system.

**Knowledge Transfer Statement:**

The results from this study provide an initial first step for those interested in exploring and working toward the paradigm shift to preventive focused dentistry. We also hope that these findings will encourage more research exploring the complex relationship among dental stakeholders, with a view to overcoming the barriers. In particular, these findings may be of use to dental public health researchers, dentists, and policy makers concerned with the prevention of oral diseases.

## Introduction

Oral health and oral health care services share many of the challenges with general health and health care (Sheiham and Watt 2000). Spending on oral health care for treatment of oral disease across the European Union is estimated to be close to €79 billion per annum, with the majority focused on restorative treatments (Patel 2012). Dental caries, gum disease, and tooth loss are all preventable conditions. Despite evidence for the proven effectiveness of common oral disease prevention, many dental care systems remain treatment oriented rather than prevention oriented (Garcia and Sohn 2012). This maintenance by health care systems facilitates the treatment of oral diseases over prevention (Chestnuttet al. 2009). Health promotion and primary prevention as health care delivery tools provide better oral and general health outcomes than treatments alone (Olsen et al. 2009). Indeed, there is evidence that chairside oral health promotion is the most effective approach, with promotion that facilitates fluoride use being particularly effective in reducing caries (Kay and Locker 1998). Furthermore, Carr and Ebbert (2012) showed that behavioral counseling with an examination led to an increase in tobacco abstinence rates.

Understanding the requirements of a paradigm shift toward a preventive oral health care system should be the first step in delivering quality health care. However, this has not received much attention to date. Broadly, oral health prevention given by a dental professional includes fluoride varnish, placing fissure sealants, prescribing high–fluoride content toothpaste, providing oral hygiene advice and instruction, and providing dietary advice (Public Health England 2017). Patients’ oral health self-care could include good oral hygiene practices (regular toothbrushing, healthy diet/lifestyle, and regular dental attendance).

Six systems have been described for the provision of oral health care in Europe (Widström et al. 2004): Nordic, Bismarkian, Beveridgian, Southern European, Eastern European, and hybrid (publicly funded [free] oral health care for some and/or all children but largely private provision for adults). There are wide variations among countries across Europe regarding the structure and delivery of oral health services to patients (Widström et al. 2004), by social, cultural, and geographic differences. Exploring these differences affords the opportunity to gain insight into the scope for improving health system design to best support chronic disease prevention and the opportunity to share good practice regarding which system features promote a preventive paradigm.

Health care systems are complex and exist on a microlevel (chairside) through to a macrolevel (professional, political, societal, and international). Identifying existing structures to understand the stakeholder ecosystem, such as networks of service commissioners, health care organizations (e.g., insurers), providers, and patients, may help us understand the delivery of prevention, as well as the attitudes and behaviors toward prevention in oral health care.

This research was conducted within a larger Horizon 2020 project: ADVOCATE–Added Value for Oral Care (Leggett et al. 2017). The project involved 6 countries representing 5 oral health care system designs: Denmark (Nordic), Germany (Bismarkian), Hungary (Eastern European), Ireland (hybrid), the Netherlands (Bismarkian), and the United Kingdom (Beveridgian).

This research aimed to explore the barriers and facilitators to the prevention of oral diseases as perceived by dental teams, dental policy makers, insurers, and the general public across 6 European countries. The triangulation of the 4 stakeholder groups within the same research study is unique and is key to identifying ways to achieve a paradigm shift in prevention and health promotion. This research was one step within a larger research goal of developing questionnaires to explore patients’ and dentists’ attitudes toward prevention across all 6 countries.

## Method

### Design

Native-language semistructured one-to-one interviews and focus groups were conducted with dental team members, insurers, dental policy makers, and the general public in Denmark, Germany, Hungary, Ireland, the Netherlands, and the United Kingdom. Ethical approval was granted from the Dental Research Ethics Committee at the University of Leeds (051115/HL/182), the University of Heidelberg, the University of Copenhagen, University Collage Cork, Semmelweis University Hungary, and ACTA Amsterdam (2017.081). The research was undertaken in full accordance with the World Medical Association Declaration of Helsinki (version 2008). A descriptive thematic analytic approach to data collection and analysis was undertaken (Braun and Clarke 2006; Nowell et al. 2017).

### Materials

Topic guides for each stakeholder group were developed based upon the findings of a systematic review exploring barriers and facilitators to prevention in oral health care. Participants were guided on what prevention might include to ensure that they considered professional advice, prescriptions, and treatments that the dental team could offer. Semistructured interviews were piloted in the United Kingdom to refine understanding and relevance of the questions. Questions were translated into the language of each country and checked for consistency and accuracy in meaning posttranslation. Where questions were changed or became ambiguous after translation, the researchers worked on a suitable alternative in keeping with the original question.

### Procedure, Participants, and Recruitment

Participants were recruited purposefully and opportunistically by local researchers in each ADVOCATE country through a variety of approaches. Recruitment via adverts, emails, and social media targeted a convenience sample of the general public and dental teams. Policy makers and insurers were identified through publically available organization information and personal contacts. It is important to note that, due to the public structure of the NHS (National Health Service), those in the insurer group from the United Kingdom were mainly representatives from the health care organization involved in commissioning dental services. Interviews and focus groups were conducted between March 2016 and September 2017 by a researcher in each country. The researchers were native speakers and trained to undertake qualitative interviews. The interviews and focus groups were undertaken by a local researcher in each country. H.L., an experienced UK qualitative researcher and psychologist not known to the participants in any other capacity, was present for at least 50% of the interviews/focus groups in each country to ensure consistency. Interviews/focus groups were undertaken at a host institution or another convenient venue or via the telephone. Informed consent was obtained prior to interview/focus group commencement.

### Analysis

Interviews and focus groups were transcribed in their original language, with names removed to ensure anonymization, and then translated into English by native-language speakers in each country who were bilingual with a high level of English proficiency. Translated transcripts were then checked by a native English speaker, and any queries regarding the English translation were discussed and resolved. Thematic analysis was undertaken, as it offers a flexible approach to identifying, analyzing, and interpreting themes within data (Braun and Clarke 2006). A deductive approach to analysis was undertaken, focusing on barriers and facilitators to prevention. As these findings were used to inform the development of questionnaires for patients and dentists, the analysis remained at the semantic level and focused on exploring what participants stated as their perceived barriers and facilitators to prevention, without deeper analysis of their underlying thoughts, beliefs, and conceptualizations (Braun and Clarke 2006).

All transcripts were coded in NVivo Version 12 (QSR International) by 1 researcher in relation to barriers and facilitators. After initial coding, 16% of the transcripts were double coded by a second researcher, and any discrepancies were discussed and resolved through consensus. Following this, an iterative process began that involved reviewing and revising the codes and themes with input from the researchers in each country. Development and refinement of themes that pertained to barriers and facilitators to provision of oral health care prevention were achieved through an iterative method of constant comparison, which identified the similarities and differences across stakeholder groups and countries to ensure that different perspectives were represented (Braun and Clarke 2006). Data triangulation was incorporated into the analysis to gain multiple perspectives and validation of data from patients, practitioners, and policy makers in each country. Continuous research team discussion of themes and the use of negative case analysis to challenge the emerging themes (a “negative case” is a less dominant or opposing theme/pattern; Allen 2017) supported the understanding of each theme and enabled development and refinement of key themes.

## Results

Across the 6 countries, 13 focus groups and 58 interviews were undertaken with a total of 149 participants (Table 1).

**Table 1.** Number of Participants from Each Stakeholder Group in Each Country.

	Stakeholder (Code)					
Country (Code)	Dental Teams (DEN)	Policy Makers (PM)	Insurers (INS)	General Public (GPUB)	No. of Interviews (Focus Groups)	Total Participants
England (UK)	6	10	5	11	18 (2)	32
Ireland (IRE)	5	1	4	11	13 (1)	21
Denmark (DK)	12	8	1	8	10 (2)	29
Hungary (HU)	7	7	0	7	7 (2)	21
Netherlands (NL)	7	4	16	5	4 (4)	32
Germany (DE)	2	3	1	8	6 (2)	14

Five themes emerged that were both barriers and facilitators to prevention (Table 2): 1) dental regulation, 2) who provides prevention, 3) knowledge and motivation, 4) trust, and 5) person-level factors. Each theme was touched on in all countries; however, cross-country differences were seen with regard to the magnitude of the theme. Quotes from participants use the following nomenclature: country abbreviation.stakeholder group.participant number (e.g., UK.DEN.1).

**Table 2.** Themes and Subthemes.

Theme	Subtheme
1: Dental regulation	Mouth-body divide and isolation
	Monitoring and quality assurance
	Funding and remuneration for preventive services
	Guidelines and contracts
	Education
2: Who provides prevention	Dentist’s perceived role
3: Knowledge and motivation	Frustration with patients
	Dentist knowledge
	Patient knowledge and motivation
4: Trust	Transparency in the system
	Dentist-patient relationship
5: Person-level factors	Sociodemographics
	Irregular access
	Cost to patients
	Dropping out of the system

### Theme 1: Dental Regulation

#### Mouth-Body Divide and Isolation

Oral health care was seen as being isolated from the policies and decisions made relating to general health care and deemed to be “not on their priority list (prevention)” (UK.INS.3). Participants thought that the mouth was viewed independently from the body. For them, this was reflected in the structure of oral health care and the systems’ attitudes to dentistry and its role within health care. It was perceived that if this was resolved, it could encourage a greater focus and importance to be placed on oral health care. This was discussed in all countries by the policy makers, dentists, and general public. However, this was not mentioned by the general public in Hungary.

UK.PM.6: I think it’s about prioritization to be perfectly honest. I think prevention isn’t necessarily difficult. I don’t think it’s necessarily expensive. I just think it’s not necessarily a priority and I think making those linkages at individual level between preventive activity and disease.

#### Monitoring and Quality Assurance

A chief barrier discussed in the United Kingdom, Denmark, the Netherlands, and Hungary was the impact of not being able to measure whether prevention had been conducted and was effective. The lack of effective monitoring meant that dentists viewed there to be “no incentive” (DK.DEN.1) for them to provide prevention. They thought that any activity measurement needed to accurately reflect the time that they spent with patients. Currently, they did not feel incentivized to spend longer with patients, since they are paid the same no matter how long they spend delivering prevention to patients. Policy makers believed that dentists did not value or want their activities to be assessed on quality and therefore were likely to be resistant to any change that addressed this; this in turn made it harder to secure a professional standard for preventive care.

NL.PM.2.P4: That is something that makes transparency very difficult because how do you want to achieve transparency when they don’t even record their own work.

#### Funding and Remuneration for Preventive Services

Funding was seen as a barrier in all countries, particularly in Denmark, Hungary, and Ireland. Dentists felt as though funding deficiencies negatively affected improvements in the awareness and understanding of prevention in patients and dentists and that investing in preventive activities would lead to long-term gains. There was a negative perception from dentists regarding the role of commissioners/budget holders in distributing resources; they perceived a lack of will from policy makers to spend the money on prevention. There was a cross-countrywide view from policy makers of “What are we paying for?” Such a view may discourage future spending and imply a lack of transparency regarding value for money.

DK.DEN.4: A redistribution of all these subsidies you could actually, within the existing small budget we have, have plenty enough money to fund lots of prevention. But there is a lack of political will. And the will is not lacking for such changes from the dentists side of the table.

Participants spoke of how the 2008 recession in Ireland negatively affected funding available for prevention and the ability to provide large-scale prevention through outreach programs. Reduced resources meant that treatment became a funding priority and funding for prevention less important. The Irish stakeholders saw prevention as providing a return on investment to the government in the long term; a focus on prevention would result in less future treatment and therefore less money spent on oral health care.

The public sector dentists in the United Kingdom and Ireland did not perceive there to be any incentive to provide preventive advice or treatment over restorative treatment. Dentists thought that they are not properly remunerated for providing prevention; the pervading view was “You don’t get paid for it” (UK.DEN.3). Dental team members in the United Kingdom and Ireland believed that they should be paid additionally for the time spent on prevention. This perceived lack of remuneration for prevention was seen as an influential barrier to more prevention being provided: “Financially it’s better for us to be doing more complex treatment, charging patients more and making more” (IRE.INS.2). In Hungary, Germany, and the Netherlands, dentists felt as though they were not well paid to deliver prevention. The increased financial return for restoration as compared with prevention negatively affected dentists’ perceptions toward prevention.

HU.PM.5: I think that if the dentists have enough time to treat the patient and it is financially supported, the dentists will be more likely to do preventive interventions.

DE.PM.2: Dentists providing preventive services need to be compensated not punished.

NL.PM.2: You are being stimulated in doing as much operations as you can and especially the operations that are well paid and I got the feeling that prevention activities are not well paid so to get a maximization of the turnover it is wise to do curative operations.

DE.DEN.2: Some dentists might feel that it’s not financially interesting for them. I mean we make way more money with our replacement services. We could neither maintain the current staff nor the current location, if it weren’t for these high-paying, often private, services.

#### Guidelines and Contracts

Participants felt as though guidelines had less of an impact on dental practice than they could have and were not always adopted. There was also the feeling that guidelines were implemented top-down, with changes sometimes occurring without notification or warning. In the United Kingdom, guidelines and contracts were seen as “vague” and implemented “without approval and without proper thought, proper process” (UK.DEN.1). Furthermore, the United Kingdom’s system structure was criticized, with the dental team reporting feeling unsupported by the NHS dental commissioners, as if they were on a treadmill to meet patient quotas set within their contracts. They saw this as not facilitative to spending time with the patient and delivering prevention.

In Hungary, dentists saw guidelines as being too long and complicated and not focusing on prevention. In Ireland, the dentists saw dental council guidelines and the fact that continuing professional development was not mandatory as barriers. Dentists in Denmark felt excluded from guideline development; they did not feel ownership or have clarity on how the guidelines were applicable to their practice. Policy makers perceived dentists as wanting to maintain their autonomy rather than having concerns with the guidelines or their implementation. In Germany, dentists and those representing the dental association stated, “We are not really involved that much [in the development of guidelines]” (DE.PM.1). They thought that the guidelines were either too deep or too vague, and this affected whether they were used. Policy makers in the Netherlands spoke of how difficult it was to apply guidelines in real-life clinical situations; some policy makers asked, “How could you make something standard in an environment that isn’t standard at all?” (NL.PM.3). Dutch dentists spoke positively of the availability and accessibility of guidelines but felt that they were sometimes unusable due to the length and clarity. The policy makers believed that it was hard to implement or ensure compliance with guidelines.

Despite these barriers, individuals in each country discussed an increased focus on prevention regarding regulation as compared with the past. The stakeholders could see how change was underway and that dental care was on the right trajectory, even though further change was still required.

DE.PM.1: Overall I think the change in the minds of the people is happening though. The awareness of the importance of prevention is there. Prevention in dentistry for the most part is already working very well.

#### Education

Individuals in Denmark, Hungary, Germany, and Ireland felt that prevention in education was not given a strong-enough focus. They suggested that treatment had the strongest focus and that prevention was added in as something that was required rather than something truly valued. In Denmark, academic policy makers thought that although prevention was taught, teachers did not really value it, thus influencing the value that students placed on it. UK dentists believed that less importance was placed on the delivery of prevention, especially in practices that did not value or prioritize it: “Even your practice as a whole might think ‘Well it’s a waste of time!’” (UK.DEN.3). This culture was seen to influence newly graduated dentists working in practice; often, there was a discrepancy between what is taught in university and how prevention is practiced in reality.

### Theme 2: Who Provides Prevention

As clinicians, many dentists thought that treatment was more interesting and the reason why they had become a dentist. Prevention was often seen as being not “fun” or “sexy” (DK.DEN.1.P2) and lacking “kudos” and being a “waste [of time]” (UK.DEN.3) as compared with treatment.

UK.DEN.1: And that’s not exciting so therefore I think the dentist shouldn’t be doing that, it should be the other . . . ancillary staff, you know the other care professionals, you know like qualified nurses and therapists.

German and Hungarian dentists believed that prevention was sometimes seen as being overemphasized and “utopic” (DE.DEN.2) and that prevention is not always appropriate to all patients.

DE.DEN.2: Yes, many problems can certainly be prevented if we pay the necessary attention. However there will always be cavities, gum problems, rotting teeth etc. We will never be able to prevent everything.

Skill mix was seen as a facilitator to prevention in Denmark, Hungary, the Netherlands, and Germany, where value was placed on the importance of having hygienists to deliver some preventive advice and treatments. Dentists appreciated the workload that other team members took on, as it freed them up to concentrate on complex treatments. They also perceived hygienists to have better knowledge, patient rapport, training, and remuneration to deliver prevention.

NL.DEN.1.P3: [Hygienists] are . . . very good in giving advice more friendly. But anyway they bring it in an understandable way to the patients.

HU.DEN.1: A dental hygienist can devote more time and attention to a patient and to prevention, and they might be better at it. If there was a dental hygienist in every practice, it would count a lot. I think this works much better in Western Europe.

DK.PM.7.P1: They need to replace some of their dentists with dental hygienists, because it needs to be lifted by another group of personnel. So it needs to be organized and administrated by dentists, but the actual execution is much more a job for the dental hygienists and well-educated dental assistants, I would say.

However, dentists in each country discussed how they recognized the need to provide preventive advice and treatment and that, regardless of the aforementioned issues, it was their role to provide it. Furthermore, many dentists believed that in recent years there had been an increased priority and focus placed on prevention within the checkup.

IRE.DEN.1: It’s good you know that we focus on prevention. . . . I think it’s always good to keep reinforcing that message ’cos you know there are various different initiatives that are in the pipeline which are always good to kind of keep changing it and keep everybody interested in prevention because as you know a clinician over time you kind of get jaded if you give the same advice so it’s nice to mix it up to give it in a different way you know.

### Theme 3: Knowledge and Motivation

#### Frustration with Patients

Dentists in all countries were put off providing preventive advice as they experienced difficulties in motivating their patients to change. They reported frustration with repeatedly giving preventive advice and there being no change in behavior or any positive impact on patients’ oral health. Some dentists perceived that patients do not always want to know about prevention and how to look after their mouths, and they noted that such experiences were demotivating, making them feel as though providing preventive advice was a waste of time as it was “falling on deaf ears” (UK.DEN.3). Alternatively, German dentists (and patients) suggested that patients do know about prevention but do not want to change. Another view expressed by dentists in Hungary was that patients see prevention as an “additional burden” (HU.PM.4), are lacking self-motivation, do not value prevention, or do not understand why purchasing toothbrushes or toothpastes should be a personal cost.

DE.DEN.1: Most people are well aware if their lifestyles are unhealthy. . . . Sometimes you can give them small tips on how to minimize the negative consequences. . . . However it’s not like you can really make people stop smoking.

Dutch dentists additionally reported that it was challenging to gauge patients’ knowledge and engagement to provide the right amount of information at the right level.

In general, dentists, insurers, and policy makers in the United Kingdom, Denmark, the Netherlands, Hungary, and Germany thought that patients did not take responsibility for their oral health or listen to preventive messages, and this was key to their lack of motivation to behavior change. Some patients were also seen as not listening to the preventive advice until it was too late and they were in pain.

UK.DEN.3: One or two groups of people who are like that. It’s you’re the dentist. It’s your job to fix everything. . . . There’s no onus on them it’s all you, you, you!

UK.DEN.1: At the end of the day I think ultimately the other thing is, prevention is all about compliance. The patients comply with the recommendations.

Dentists perceived that motivated patients had positive outcomes from preventive advice and treatment. Patients, dentists, and policy makers in the United Kingdom, Denmark, the Netherlands, Germany, and Hungary spoke positively of “habit forming” (UK.GPUB.1.P2) for patients, whereby healthy habits were formed usually in childhood around dental attendance and oral health maintenance. Despite these challenges, many dentists felt that it was their role to repeat messages but that prevention was not just their responsibility and that more effort needed to be made by the patient.

#### Dentist Knowledge

Dentists in the United Kingdom, Hungary, and the Netherlands discussed that it was often difficult to transition to a more preventive focus as their training focused on treatment. This was evident in the United Kingdom, Denmark, the Netherlands, and Hungary, where dentists felt ill-equipped regarding preventive advice.

UK.DEN.4: I think that’s quite a scary place for some of the older dentists to go because they aren’t used to working in that style and so I think that . . . there’s a training need I think, but whether you would get the dentists who need it to turn up to the training courses? ’Cos . . . for them it’s admitting that they don’t have this new skill set and why that new skill set’s important.

#### Patient Knowledge and Motivation

In all 6 countries, the general public had a relatively good understanding of its oral health care and reported having good oral health practices. Patients in all countries were very receptive to preventive messages, valued advice, and believed that it was beneficial. However, patients wanted more information about their oral health and how to take better care of their mouths. They felt as though they currently were not well informed and had differing views on what prevention entailed.

UK.GPUB.1.P2: I think personally I would say prevention is . . . just a number of things. Like your diet. And also em . . . ensuring that you are brushing your teeth twice a day . . . yes.

DE.GPUB.2.P4: It’s the usual things. Don’t drink sugary drinks, don’t eat sweets, etc. Sugar-free gum.

IRE.GPUB.1.P1: Brushing, flossing maybe flossing, brushing twice a day once in the morning and once in the evening mouthwash in the mornings.

DK.GPUB.1.P6: It requires that you understand the reasons behind why you do as you do.

Patients also viewed dentists as not being interested in prevention and not giving enough (or any) advice in the checkup. Conversely, policy makers and dentists in all countries believed that there was a lack of public education related to oral prevention.

DK.GPUB.1.P6: I have experienced my dentist telling me something didn’t need doing now and then at the following visit, it did need to be done. I couldn’t help to suspect that it might had been okay to wait even longer. . . . So to obtain clarity about what is it all about and be sure that it has been understood why the treatment may be postponed is very important. Treatment is of course voluntary, but the conversation about why it is suggested and an understanding of why it is needed is important.

Patients in the United Kingdom, Denmark, Ireland, Germany, and the Netherlands found change difficult—in particular, long-term sustained change. Some patients agreed that the advice would not make them change; however, some perceived that they were actually trying to change their behavior and valued the advice given.

UK.GPUB.1.P5: I think there’s that initial guilt thing isn’t there? . . . Then maybe for the next 2 weeks or something you find that 5 minutes in your day to do your flossing regularly and then it sort of slowly edges off?

A barrier to making long-term sustained changes was the lack of immediate reward that the patient gets. Oral health improvement may take time, with results not visible or known to the patients until they return to the dentist. This lack of positive immediate reinforcement meant that the change was not rewarded or reinforced and therefore not maintained over time.

UK.PM.4: They have to be informed by somebody else. There’s no feedback mechanism unless you’re talking about bleeding on brushing I suppose. You say, “Well just keep on brushing and it will get better.” But a lot of things about decay you don’t see it!

### Theme 4: Trust

#### Transparency in the System

Members of the general public from Hungary, the Netherlands, Denmark, and Ireland thought that there was little transparency between dentists and patients. They believed that dentists were in control and patients had to trust the dentist, without having the knowledge or ability to know if they were being correctly or fully informed or overtreated or mistreated. Patients in the Netherlands and policy makers in Hungary also believed that this lack of transparency between dentist and patient was unique to dental care. In addition, the Hungarian policy makers believed that dentists liked the lack of transparency, as it gave them more control.

NL.GPUB.1.P3: I don’t have enough knowledge to be able to say if what you’re saying is right or not.

DE.GPUB.1.P6: If a doctor tells me, you need this, I trust them. I know nothing about these things. I think there are plenty of dentists that try to make the most money with patients.

UK and German patients felt as though they received mixed messages and inadequate clear take-home messages, perceiving dentists to “contradict themselves frequently” (DE.GPUB.1.P5). There was a conflict between guidelines and the message given in advertising campaigns, particularly the messaging related to sugar. Most knew that sugar causes tooth decay; however, the impacts of “healthy” sugars (e.g., fruit and fruit juices) were often not conveyed to patients or not understood by them.

#### Dentist-Patient Relationship

All countries felt the importance of the dentist-patient relationship for patient motivation. Patients in Denmark and the United Kingdom mentioned a rushed consultation and feeling like a “money-making machine” (DK.GPUB.2) for their dentist. Those in Ireland, Denmark, and the Netherlands reported often not feeling listened to (IRE), valued (DK) or trustful of their dentists (IRE, NL). Dentists in the United Kingdom, Ireland, and Germany were criticized by patients for being money focused. Dentists acknowledged that they were “still often perceived in a negative way” (DE.DEN.1).

Dentists recognized the importance of how they spoke to patients and the importance of their patients trusting them. They recognized that the message needs to be given in a way that does not blame or lecture patients. How the message is given influences the uptake of the message: it needs to be delivered in a way that the patient is receptive to, and it needs to be tailored to each patient.

DE.GPUB.1.P2: I mean I don’t find it annoying or inappropriate either. However if someone tells you that you have to brush and floss like a thousand times a day or you will lose all your teeth in the next ten years, it kills all my motivation.

Positively, patients felt as though their needs are more likely to be taken into account nowadays than in the past and they are more likely to be kept informed.

DE.GPUB.2.P3: I think it’s already much better. They communicate more. Ask you how you feel, pain and so. I think especially younger ones have been trained to be more careful.

### Theme 5: Person-Level Factors

#### Sociodemographics

Patients from a socially disadvantaged background were viewed by all groups as being more likely to be influenced by cost, to have lower oral health knowledge, have lower prioritization and awareness of oral health, and to engage in risky behavior. The most vulnerable groups access dental services less frequently than more affluent patients; this was recognized in the Netherlands, Denmark, the United Kingdom, and Ireland. Even if cost was not always an issue, there appears to be a complex interplay among priority, motivation, and attendance for all patients.

HU.DEN.7: We can see the correlation, those who are in a worse financial standing will not visit us, not just because they cannot afford it, but because they cannot pay attention to it, since they have other problems. On the other hand, if something is free of charge, many people think that it is also bad or it does not matter. But those who give money for it, also pay attention to it and would like to know what is happening to them.

DE.PM.1: The traditional explanation is that poorer people, people with lower education status engage in riskier behavior such as smoking, drinking [alcohol] or unhealthy nutrition.

#### Irregular Access

Irregular attendance was seen as a barrier to receiving prevention; these patients were more likely to require symptomatic treatment due to postponing appointments until treatment was required and to have worse long-term outcomes. Symptomatic attendance reduced opportunities to receive ongoing, regular preventive advice and treatment. Continuity of care was seen as challenging for such patients. Without an ongoing conversation on prevention, there was often a lack of continuity and development of the preventive message, which could have otherwise increased the health literacy for some patients. Some dentists believed that it was too late or not the right time to discuss prevention with irregular attenders, believing them to be less receptive to preventive messages.

HU.DEN.3: They get the urgent care automatically and then they do not come back to dental hygiene treatment or to further necessary treatments. Then if an acute problem occurs, they appear again and get the urgent care automatically. I think this is also a major barrier to preventive care.

DE.DEN.2: The bigger challenge is that people just don’t go to the dentist. Those that would probably need it the most.

In the United Kingdom, access to NHS dentistry was seen as a barrier to getting the preventive message across. Patients spoke of “being thrown off the books” (UK.GPUB.1.P4) and having difficulty seeing an NHS dentist. Some patients in the United Kingdom and Ireland also reported that they did not understand how their dental care system operated. Similarly in Hungary, patients stated that they found it difficult to secure an appointment quickly and at a convenient time for them.

IRE.INS.4: Basically the key word is awareness and education is another key word that people don’t really know what they’re entitled to or what they are covered for.

#### Cost to Patients

The high cost of dentistry was recognized by policy makers in all countries as having a negative impact on patient attendance and choosing a treatment. In Ireland, medical card entitlements were considered as a cost-related barrier and as facilitating treatment over prevention since those with medical cards are entitled to “only have 2 fillings in the year but you can have as many extractions as needed so they’re telling the person have as many teeth pulled and have a denture put in there rather than have a filling done which is just ridiculous” (IRE.DEN.3). The focus on treatment over prevention suggests that less value is placed on prevention, and it is not incentivized to patients. Activities such as a scale and polish are also no longer funded through the medical card scheme; this appointment was historically used to disseminate health care messages.

Patients in Hungary and Denmark reported the cost of dental care as a barrier to attending the dentist. The impact of cost as a barrier was influenced by how much the patient valued oral health care rather than cost being a barrier alone. Those seen as not being able to afford oral health care were often stereotyped as those who did not care, value, or prioritize their oral health. Patients from the United Kingdom, Demark, the Netherlands, Ireland, and Germany were perceived as not wanting to pay for advice. Furthermore, attitudes to the dentist tended to prevail through generations: if parents did not value their oral health, did not attend the dentist, or had a dental phobia, then the child would likely behave in a similar way, as the habit (i.e., dental attendance) would be less likely to form or be reinforced.

UK.GPUB.1.P6: Cycles, you know cycles of poverty. Cycles of deprivation. Haven’t seen their parents doing it. Wasn’t important.

#### Dropping Out of the System

Dutch and Danish stakeholders discussed issues related to no “smooth transfer” (DK.PM.1) between systems; as a consequence, patients often dropped out of the system, usually between the state-funded child services and the fee-paying adult systems. This is likely due to the cost but may be due to patients having to take on responsibility for their own health in adulthood. Participants also felt as though there was a lack of support for accessing care or appropriate services for the elderly, regardless of how it was paid for.

DK.PM.7: The biggest challenge we have in Denmark, when we think about prevention, is that there is not good enough transfer between the various dental care systems that we have. We have too large dropout. . . . It is only around 70% that are transferred to the adult dental care, whereby some important prevention lost.

NL.PM.2 : But the problem is that the low socioeconomic and vulnerable groups like the elderly are the ones who take less oral health care. So the ones with the lowest income need the most care.

## Discussion

These findings provide a unique insight into perceived barriers and facilitators to prevention for the general public, dental teams, policy makers, and insurers across 6 European countries. Key barriers to prevention were identified: the way that each system is regulated, dentists’ ability and desire to deliver prevention that actually leads to improved oral health, and patients’ engagement with oral health care messages.

Underlying the 5 themes is an atmosphere of “blame attribution” (Molnos 1998), whereby each group shifts responsibility to other groups. The policy makers viewed others with policy remits as not prioritizing prevention, dentists as not delivering it, and patients as not taking responsibility for their own oral health. Dentists were dissatisfied with the policy makers and insurers for how dentistry is regulated, and they saw patients as not wanting to take responsibility for their oral health. Patients viewed dentists as not providing clear preventive advice specific to them. This seems to create a complex culture of blame that results in inadequate prevention being delivered. This responsibility shifting aligns with the existence of between-group suspicions, whereby negative perceptions of other groups of stakeholders were often held (Bartling and Fischbacher 2011). This can be seen throughout the themes in which one group of stakeholders holds negative perceptions about how another group feels or acts toward the domain of interest. The presence of such attitudes reiterates the importance of fostering shared decision making between patients and their dental providers and the significance that this could have for both parties. Indeed, shared decision making was highlighted in the United Kingdom by the General Dental Council in a recent guideline that addressed this issue for patients and practitioners (Collaborating Centre for Values-Based Practice in Health and Social Care 2019). It appears that there would be value in a similar approach to effective engagement between policy makers and providers on the provision of preventive dental care.

Dentists voiced frustration with the usability of guidelines on prevention and the top-down expectation that guidelines and system changes should be adopted by them with little warning or option. Previous research showed that guidance uptake was improved if a strong professional backing was attached to it with clear, clinical context and relevance (Sheldon et al. 2004). This suggests that more collaboration among the dental team, commissioners, and policy makers could work to positively influence relations and foster greater focus on prevention (McNicol et al. 1993; Spallek et al. 2010).

Inadequate reimbursement and budget cuts were also thought to hinder the delivery of more prevention, further compounded where prevention is seen as less of a priority. There were dissimilar perceptions from those paying for and those providing preventive care regarding fair reimbursement and how such dental activity should be monitored. A lack of explicit and fair remuneration for prevention was seen as a barrier to the delivery of more prevention. This was less of an issue in countries such as Denmark, the Netherlands, and Germany, where prevention is remunerated as an item of care. However, dentists still felt that this fee was not enough for the time needed to conduct meaningful prevention.

There is conflicting evidence on the effectiveness of financial incentives alone leading to the provision of more prevention. Research suggests that offering a fee can lead to an increase in the provision and delivery of prevention services (Clarkson et al. 2008). Contrary opinions have also been suggested: although financial incentives encourage change, their impact lessens with continuous exposure (Gnich et al. 2018). While monetary incentive-based systems may offer extrinsic motivation to perform a task, they have been shown to reduce individuals’ intrinsic motivation to perform a task (Grytten 2017). Intrinsic motivation is often seen as being more powerful than extrinsic motivation and is more likely to be associated with increased job satisfaction (Goetz et al. 2012). There could be the concern that by placing a greater emphasis on payment for prevention that the system shifts to an environment whereby dentists do something only because they receive payment for it rather than because it is their professional obligation and duty to provide it (Voinea-Griffin et al. 2010). Encouraging behavior through payment for that behavior may increase its likelihood but may not increase individuals’ value or attitudes toward it. Consequently, it could be argued that a greater focus on education surrounding prevention and behavior change, coupled with a fairer pay scheme, may aid the delivery of prevention through dentists’ greater understanding and knowledge of prevention and a satisfaction with the payment received for time spent on this (Suga et al. 2014). However, without prevention being a priority for the dental care system and without effective quality assessment, providing a greater financial incentive to dental teams was hard to justify for the policy makers.

Perceptions around who is best placed to deliver prevention revealed interesting insights. Some dentists felt that prevention was not their role. This perception prevailed across the counties, which suggests that this may be the mind-set of being a dentist, rather than a by-product of the system in which they work. The dentists who felt that prevention was not their role were also those who 1) believed that it had little impact on patients’ health outcomes and 2) had experienced frustration and demotivation due to patients not changing their behavior or adopting the advice. Previous research supports the positive impact that perceived role can have on delivering prevention. Gnichet al. (2015) showed that rates of fluoride varnish application were positively associated with dentists’ perception that its application was part of their professional role. Motivation is also important here: Gnich et al. and Elouafkaoui et al. (2015) both found that dentists were more likely to provide fluoride varnish if they felt motivated to do so and had a positive attitude toward the activity. Ultimately, those who do not view prevention as their professional role are likely to be less motivated and have weaker positive attitudes and self-efficacy toward delivering it. By not providing prevention to those whom they perceive to not value the advice, dentists may be fueling and widening the knowledge gap among members of society and increasing oral health inequalities (Gordon et al. 1999). As such, it is likely that those who are most in need are the ones who are less likely to attend the dentist and receive prevention advice and treatment (Donaldson et al. 2008).

The effective use of skill mix was a facilitator for prevention in countries such as Denmark and the Netherlands, where hygienists are being utilized more within their systems. This supports previous research suggesting that more effective use of hygienists may facilitate the delivery of prevention through these professionals (Brocklehurst and Macey 2015). Indeed, Öhrn et al. (2008) found that patients had less negative attitudes toward dental hygienists in comparison with dentists. However, issues remain regarding the cost to the patient of utilizing these services, their satisfaction with care from other members of the team, as well as balancing the dentist-hygienist power relationship (Dyer and Robinson 2006; Cannell 2018). There appears to be a fine balance between dentists 1) valuing the hygienists’ time and better skills in advice giving and 2) fearing role replacement and territory encroachment (Dyer & Robinson 2006).

The interviews and focus groups suggest that patient education and oral health awareness are lacking across Europe. Members of the general public in some countries appeared to have better general oral health knowledge and involvement in their care than others. However, regardless of their level of knowledge and involvement, there was the feeling overall that they could know more about how to maintain their oral health. In recent years, there has been an increased provision of educational toothbrushing programs for children in schools and enhanced support and contact at dental practices (Davies et al. 2011; Macpherson et al. 2019). However, there is a lack of continued knowledge or information provision surrounding oral health care information to adults. It is widely debated 1) the extent to which information giving alone leads to change (Yevlahova and Satur 2009), 2) if short-term knowledge gain translates into sustained oral health improvements (Baelum 2011), and 3) whether the message should be delivered by the dentist or another member of the team (Baelum 2011). However, the success of short-term behavioral interventions within the dental practice suggests that patients can be encouraged to change their behavior, leading to positive oral health outcomes with the correct support (Werner et al. 2016; Wide et al. 2018). Key to the sustainability of this long-term is the availability, skill set, and investment (valuing) of a dental team to provide such interventions in the practice without the external support from a research-led intervention. Unfortunately, the findings from this research suggest that these elements may be unobtainable within current care systems.

Discussions surrounding the dentist’s perceived role in prevention and patient knowledge, motivation, and responsibility all point to the need for greater dental training with a focus on understanding and supporting patient behavior change. Perhaps it is the role and responsibility of the education system to support dentists in their communication with patients and training in bringing about psychological behavior change. More targeted education that is grounded in the psychological models of behavior change could help to address dentists’ perceived role in delivering prevention and their frustration with patients not adopting the advice. Furthermore, a greater focus in education on the importance and key strategies of communication and rapport building could be useful for helping dentists build stronger relationships with their patients. This may to lead to higher patient satisfaction, adherence to advice, and better oral health outcomes (Sbaraini et al. 2012). Motivational interviewing has been shown to be the most effective approach in a clinical setting for changing patient health behaviors (Yevlahova and Satur 2009), but issues remain of how to deliver this in daily clinical practice. Addressing the way in which dentists communicate with and support patients could lead to increased patient knowledge, enhanced motivation to change, and improved job satisfaction. The challenge remains, however, of implementing this change.

### Strengths and Limitations

This research adds to the limited existing literature about barriers and facilitators to prevention by offering a wealth of information from a broad range of stakeholder perspectives, health care systems, and countries. The present research, drawing on experiences and views across multiple settings in Europe, demonstrates that the barriers to prevention are not just a feature of the health care system. The interviews were undertaken in each native language, which meant that participants could speak freely without translation worries or language issues. In addition, the translated transcripts were back-translated by the appropriate native researcher and checked to identify any confusion around meaning. The sense checking of the topic guides and the process of back-translation of the transcripts were aided by the close working relationship of the core research team and the researchers in each country, which helped to smooth out any methodological issues due to undertaking multilingual research on this scale. The fieldwork was undertaken by researchers who were not practicing dentists, which may have lessened the impact of social desirability bias for the dentists and reduced the impact of researcher bias for the patients, policy makers, and insurers.

The number of participants recruited in each group within each system was intentionally small, as the purpose of this qualitative study was to obtain an in-depth understanding across a broad range of stakeholders’ views in 6 countries. It should therefore be noted that the generalizability of the findings may be limited and that the interpretation of the data remained at a thematic and not interpretivist level. Due to the necessary processes involved in undertaking interviews in multiple languages and having them translated, the process could not be as iterative as hoped, and so data were collected through convenience sampling and completed before all transcription and translation were fully undertaken. Therefore, while data saturation was not reached (i.e., we did not sample participants following thematic analysis), thematic saturation was reached within the data, since no new themes were emerging after all transcripts had been coded and analyzed. The research may have attracted individuals with an “axe to grind” with regard to oral health care. Indeed, the participants more readily shared experiences and thoughts around barriers to prevention. However, the questions were posed in a neutral manner to avoid explicitly positive or negative responses. Furthermore, the interviews were moderated to ensure that they kept on focus and were a balanced discussion. Therefore, it is unlikely that the greater focus on barriers is due to the questions asked and is more likely to be a true reflection of these participants’ experiences. The lack of discussion around facilitators could be explained by familiarity; that is, it may be hard to discuss a facilitator within your system if it is something that you are already used to. Furthermore, the fact that similar experiences were reported across countries implies that the findings represent lived experiences in each country. However, we were unable to explore participants’ backgrounds and sociodemographic status within the interviews, so we are unable to determine whether this had any impact on their responses or the data collected. While some dental hygienists were interviewed, the majority of participants from the dental team were dentists. Future research should include a greater proportion of dental hygienists and other members of the team to further explore the role and impact of prevention from a more encompassing team perspective.

Thematic analysis was used to analyze the findings from each country and then to compare across all 6 countries together. A strength of this approach is that it allowed for theoretical freedom, as it is not tied to any individual theory or epistemological approach—thus making it a flexible research tool. However, apart from the steps offered by Bruan and Clarke (2006), there is little in the way of formalized guidelines for conducing thematic analysis, which may result in an anything-goes approach. To strengthen the analysis, codes for each country were checked with at least 1 other researcher to ensure objectivity. The themes identified in this research were used to identify important domains for inclusion in a questionnaire exploring patients’ and dentists’ attitudes to prevention. For these purposes, it was necessary to draw out commonalities among countries and to keep the analysis at a broad, overarching level to ensure that the viewpoints of all stakeholders in each country were captured. Positively this enabled a broader understanding and comparisons of oral health care across all 6 countries as a whole. However, it means that the depth of insight into each country was more constrained and that subtle country nuances may have not been identified. Future secondary analysis of these data may be worthwhile for interpretivist analysis of each country to explore how and why prevention is constructed by the participants.

## Conclusion

This research is truly novel in its approach to exploring barriers and facilitators to prevention from the perspectives of 4 groups of stakeholders from 6 European countries with different oral health care systems. The findings suggest that across all 6 countries, prevention in oral health care is hindered by a complex interplay of factors with differing perceptions on the same issues by various stakeholders. While there were some country-specific differences, the overarching barriers were evident in each country. Despite the differing structures of the oral health care systems in the 6 countries and the differing oral health statuses of the populations interviewed, there does not appear to be 1 dental health system that affords significantly greater user satisfaction or outperforms the rest. Despite the strengths and weaknesses of each system, those who deliver, organize, and utilize each system experienced similar barriers to prevention. This suggests that delivering effective prevention is more than just each individual or the structure of the system; rather, it is a complex interplay among all these elements. Furthermore, underlying the themes were sentiments of blame, whereby each group appeared to shift responsibility for prevention to other groups. To move forward, greater teamwork surrounding the commissioning of prevention is required, with a focus on positively changing the value placed on prevention by all stakeholders. Alongside this, further training for dental teams on how to foster effective behavior change may facilitate a greater focus on the delivery of prevention within oral health care. The measures outlined here could facilitate a step away from the blame culture that has developed and instead support and encourage a shared agenda, with common goals facilitating a prevention paradigm within dentistry.

## Author Contributions

H. Leggett, contributed to design, data acquisition, analysis, and interpretation, drafted and critically revised the manuscript; J. Csikar, K. Vinall-Collier, contributed to conception, design, data analysis, and interpretation, critically revised the manuscript; G.V.A. Douglas, contributed to conception, design, and data interpretation, critically revised the manuscript. All authors gave final approval and agree to be accountable for all aspects of the work.
